# Use of Cognitive Interviews to Adapt PROMIS Measurement Items for Spanish Speakers Living with HIV

**DOI:** 10.1155/2016/8340863

**Published:** 2016-02-28

**Authors:** R. Solorio, N. C. Ayala, E. Paez, A. M. Skalicky, L. S. Morales

**Affiliations:** ^1^Department of Health Services, School of Public Health, University of Washington, Seattle, WA, USA; ^2^Department of Global Health, University of Washington, Seattle, WA, USA; ^3^Owen Clinic, University of California San Diego Medical Center, San Diego, CA, USA; ^4^Group Health Research Institute, Seattle, WA, USA

## Abstract

*Purpose.* To use cognitive interviewing techniques to assess comprehension of existing Patient-Reported Outcomes Measurement Information System (PROMIS) items among Latinos living with HIV and then refine items based on participant feedback.* Methods.* Latino monolingual Spanish speakers living with HIV (*n* = 56) participated in cognitive interviews. Items from four PROMIS domains, including depression, anxiety, fatigue, and alcohol use, were assessed for comprehension. Audiotaped interviews and handwritten notes were subjected to content analysis to identify problems specific to each instrument for each domain.* Results.* The assessments from the cognitive interviews identified areas for improvement in each domain. We present data on the type of items that were difficult to comprehend and provide examples for how items were refined based on participants' and PROMIS Statistical Coordinating Center (PSCC) feedback. Six out of 48 depression items, 7 out of the 61 anxiety items, 18 out of 42 fatigue items, and 7 out of 44 alcohol use items were found to have poor comprehension. These items were refined based on participant feedback; the items were then submitted to the PSCC for additional guidance on linguistics and grammar to improve comprehension.* Conclusions.* Cognitive interviews may be used to enhance comprehension of PROMIS items among Latinos.

## 1. Introduction

There are 51.9 million Latinos in the United States making up 17% of the total population [1]. Among all Latinos in the US, 60% are US-born and 40% are foreign-born [1]. Among all Latinos in the US, 38% are monolingual Spanish speakers [1]; given this large number, it is critical that survey measures used in research and in clinical care be available in Spanish and be understood by populations with low levels of literacy. This is especially true of measures that assess conditions that often affect people living with HIV, such as depression, anxiety, fatigue, and alcohol use.

Spanish-speaking Latinos in the US may come from Mexico, Central America, South America, and the Caribbean; among these, Mexicans are the largest subgroup, accounting for 65% of all US Latinos [2]. Because there are significant phonological, grammatical, and lexical variations in the Spanish spoken by Latino subgroups in the US, cultural adaptation, including the exclusion and substitution of words or phrases, may be needed [3]. Use of a survey instrument or item bank without careful consideration of the target population's culture(s) and language use can result in poor item comprehension and its sequelae, including diminished responses rates and misleading results [4].

The translation of survey instruments for research is a complex process often involving multiple translators conducting independent forward and backward translations [5]. Conceptual and semantic difficulties can arise when translating idiomatic phrases to convey concepts that are unique to a particular region, country, or society. Even when a translation is created following rigorous methods, additional translations or refinements to existing translations may be necessary due to subgroup differences in culture and language [3, 6].

**Figure 1:**
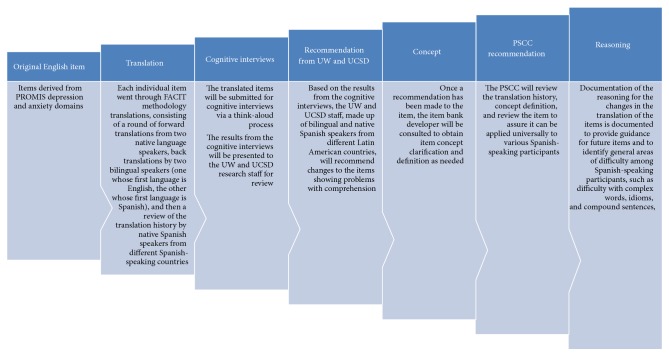
Flow diagram demonstrating the use of cognitive interview results on comprehension among Spanish-speaking participants.

**Figure 2:**
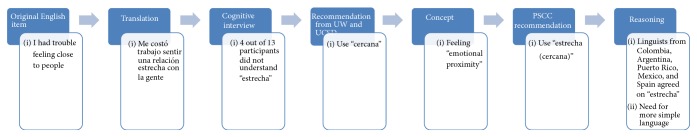
Example of the use of the flow diagram on one of the depression items.

The purpose of this paper is to illustrate how cognitive interviewing techniques can be used to improve comprehension of PROMIS I and PROMIS II depression, anxiety, fatigue, and alcohol use items with Spanish speakers living with HIV. PROMIS is a multiyear effort funded by the National Institutes of Health to create item banks for assessing patient-reported health areas across diseases and chronic conditions (http://www.nihpromis.org). We present data on the type of items that were difficult to comprehend and present examples for how we went about refining and rewording items based on participants' and PSCC feedback (Figures 1 and 2).

## 2. Cognitive Interviewing

Cognitive interview techniques were developed to improve the quality of survey data by reducing response error that can occur if questions are not interpreted in the manner they were intended [7, 8]. Grounded in cognitive psychology and information processing theory, cognitive interviewing employs the verbalization of thoughts, feelings, interpretations, and ideas that come to mind while examining survey questions [9]. In addition, respondents are asked to suggest alternate wording to increase comprehension. The use of cognitive interviewing is increasingly recognized as an important part of the formative evaluation process for questionnaire development [7, 8, 10].

Multiple approaches have been described for conducting cognitive interviews. Strategies that have been used for questionnaire design and development include the think-aloud interviews, respondent debriefing, probing techniques, and paraphrasing [9, 11]. In the think-aloud process, participants are asked to respond aloud to a specific set of questions that address the instructions provided with the questionnaire, items within the questionnaire, and response options [12]. In respondent debriefing, after a participant completes a questionnaire, an interviewer probes for specific information about what made some items difficult for the participant to comprehend [11]. With cognitive probing, participants are asked to provide information on the clarity and comprehensibility of instructions, the meaning of individual items and response choices, and the relevance of each item [11, 13]. In paraphrasing, respondents are asked to repeat a survey item in his/her own words. Often, a combination of these approaches is used [14].

## 3. Methods

### 3.1. Setting

This project constituted part of larger study that seeks to assess content validity of PROMIS depression, anxiety, fatigue, and alcohol use items in the context of clinical care for persons living with HIV who are English and Spanish speakers [15]. For this study, cognitive interviewing was used to assess comprehension of PROMIS I and PROMIS II depression, anxiety, fatigue, and alcohol use items and then refine these based on participant feedback. The adaptation of the measures described in this paper is the first step to improve comprehension of items among Latinos living with HIV who are monolingual Spanish speakers with low levels of literacy; in a future step and with a larger sample, the psychometric characteristics of the adapted instruments will need to be conducted. All procedure and consent forms were approved by the University of Washington and University of California at San Diego Institutional Review Boards.

### 3.2. Participants

Fifty-six Latinos who were monolingual Spanish-speaking adult men and women living with HIV were recruited to participate in this study (see Table 1). The participants were recruited in Seattle, WA, and San Diego, CA. The participants were recruited from two Centers for AIDS Research (CFAR) Network of Integrated Clinical Systems (CNICS) sites, including the University of Washington, Madison Clinic (Seattle), and the University of California at San Diego, Owen Clinic, and from Entre Hermanos (EH), a community-based organization serving the Latino LGBT community in Seattle, WA.

To be eligible, participants had to meet the following criteria: (a) be of Latino heritage; (b) speak Spanish only; (c) be 18 years of age or older; (d) live in King County, WA, or San Diego County, CA; and (e) meet criteria for diagnosis of depression, anxiety, fatigue, or alcohol use. The Patient Health Questionnaire-9 (PHQ-9) was used to determine depression severity, including suicidal ideation [16, 17]. Anxiety severity was measured with the PHQ-5 [18]. Fatigue severity was measured with the HIV symptom index [19]. Severity of alcohol use was measured using the Alcohol Use Disorders Identification Test [20].

Eligible participants were approached by study recruiters after their clinic/agency appointments or via telephone and invited to learn more about the study. Those willing had study procedures explained to them and informed written consent was obtained. Participants received a $25 incentive payment for completing the 90-minute interview. All interviews with study participants were conducted in a private meeting room at the clinic site or at the EH site. A trained, bilingual research assistant conducted the interviews in Spanish and each interview was recorded.

### 3.3. Instruments

We used PROMIS I and PROMIS II items for this project. Patient-reported outcome (PRO) measures use answers that patients provide to questions to produce numeric values which indicate patients' state of wellbeing or suffering as well as their ability or lack of ability to function (these are also referred to as “items”). PROMIS measures have been developed for a wide range of chronic diseases, including HIV.

For this study, we are interested in assessing PROMIS I and PROMIS II items for comprehension among Latinos living with HIV who are monolingual Spanish speakers with low levels of literacy and then refining items, based on participant feedback. We are primarily interested in using PROMIS I and PROMIS II items from three domains, depression, anxiety, and fatigue, and one subdomain, alcohol use. We examined 195 items from PROMIS I and II. Within PROMIS I, we selected 152 existing items; these items had all been previously translated into Spanish in accordance with PROMIS network standards [3, 5, 21]. These existing items had been previously tested among Latino adults [5].

We also selected 43 items from PROMIS II using the item generation methodology recommended by the PROMIS network [21, 22]. The item set that we selected contained 10 items for depression, 16 for anxiety, 10 for fatigue, and 7 for alcohol use. We translated each of these items from English into Spanish with the help of the PROMIS Statistical Center (PSC) [3], following the Functional Assessment of Chronic Illness Therapy (FACIT) translation methodology [5].

The PROMIS I and PROMIS II item banks have been developed in part through cognitive interviewing [13], a technique that provides researchers with participant input for each PROMIS item tested [11, 12, 21]. For the PROMIS I item bank, however, some items were drawn from existing questionnaires and had not undergone cognitive interview testing and some were legacy measures that were validated using other methods [23].

### 3.4. Study Design and Procedures

The described work was conducted in multiple steps. All steps were completed in serial order for the depression, anxiety, fatigue, and alcohol use items.

A semistructured protocol was developed for each scale (Spanish versions). The protocol consisted primarily of cognitive probing and paraphrasing.

For all PROMIS items, testing consisted of administering the scale items and subsequently asking individuals for the meaning of selected words or expressions. When participants did not understand a question, the intended meaning of the question was explained. Participants were also asked to suggest alternative wording or phrasing to improve the clarity of questions that were difficult to understand.

**Step 1 (definition of patient groupings by domain).**

Table 1 describes the study sample. To ensure broad representation of Latino persons living with HIV across severity levels for each domain, we defined severity levels for each domain as assessed by the clinical assessment and then selected Latinos living with HIV for each severity level to test for comprehension of PROMIS items (Table 2).

**Step 2 (cognitive interviews assessed PROMIS item comprehension for anxiety, depression, fatigue, and alcohol use).**

Prior to the cognitive interview, participants completed a domain-specific item set (e.g., only the depression items) through paper and pencil administration. We followed this with the cognitive interview, wherein we asked open-ended questions regarding response categories, time frame, item interpretation, and domain content.

A trained RA reviewed each item stem and its response with the participant. The RA began by using the standardized question (see Table 3) for each item. The RA recorded the participant's interpretation of the item and opinion on preferences in an Excel spreadsheet. We also asked participants about the extent to which each item contained information they considered most important to communicate with their provider regarding their experiences, whether the questions included all the issues they deemed important for their provider to know regarding their experience with depression/anxiety/fatigue/alcohol use, and what was missing from each list of items.

The cognitive interviews evaluated participants' comprehension of the translated item. Each PROMIS English item was taken through three main steps necessary to meet FACIT methodology requirements [5]. The interviewers first took the participants through the think-aloud process and then performed the respondent debriefing. In the respondent debriefing, participants reviewed the problem areas and identified common themes and possible solutions.

Cognitive interviews were conducted with 56 Latinos living with HIV for the first round of this project, then with 28 for the second round, and then with five for the third round. Each participant was tested with PROMIS items from one domain, using one set of instructions, and one response scale.

For the first round of item testing, we tested 195 PROMIS items. This was followed by a second round of cognitive interviews on 54 items, and a third round on 21 items. All interviews were audio-recorded.

### 3.5. Qualitative Analysis

The audiotaped interviews were reviewed by three of the coauthors. Handwritten notes taken during the interviews were also examined to provide additional clarity and detail. The cognitive protocol that was developed to guide the interview process by identifying in advance the specific words and phrases in the surveys to be examined also facilitated data analysis by providing a structured framework to systematically review the data and to analyze the interview content. Content analysis was conducted to identify, code, and categorize primary patterns of data. Data were coded by three coauthors, using Atlas.ti. Review of the data revealed trends in participants' level of understanding about the wording of specific items in each domain. In addition, participants' suggestions regarding alternative wording and phrasing were examined. The same procedures were used to analyze all domains.

We extracted participant quotes from cognitive interview transcripts and sorted them with each corresponding PROMIS item to assess the level of comprehension of the concept being portrayed in each existing item. If more than one respondent had difficulty understanding the concept of an item or had an understanding that was different from what was intended to be conveyed, the researchers reviewed the item for potential wording changes. PROMIS I items had already gone through one round of cognitive interviewing with five-to-ten Spanish-speaking participants. We did not anticipate finding many PROMIS I items that produced difficulty in comprehension. Nevertheless, this step was an important one in assessing the validity of the domains for this specific population.

The interview guide we used for the cognitive interview of the PROMIS items is shown in Table 3. We documented difficulties in comprehension, misunderstanding, or uncertainty in the meaning of items in a computer spreadsheet. We also captured information extracted from the audio recordings and participants' suggestions for translation changes for difficult-to-understand items. The full research team reviewed the final spreadsheet summary of participant feedback for each item.

We submitted items to the PROMIS Statistical Coordinating Center (PSCC) for grammatical, linguistic, and FACIT methodology review. If we proposed wording changes, we explained the reasoning for the changes, taking into account the need for the item to be comprehended by Spanish speakers from a diverse group of countries. The PSCC team reviewed our proposed modifications and evaluated their translatability beyond Spanish. Modified items required at least five additional cognitive interviews per item to be conducted. The purpose of additional cognitive interviews was to ensure the comprehension and understanding of Spanish-speaking participants of the finalized items.

In cases where problems were encountered, the participant was asked to suggest alternative wording to help clarify the question or phrasing of the instrument to better convey the intended meaning. Subsequent interviews began by using the original wording of the question. Once the first round of interviews was completed, respondent comprehension of items was assessed for each domain. We conducted a total of three rounds of cognitive interviews for each domain; after each round, item modification was conducted based on participants' feedback; we used participants' own words or suggestions; we then sought the advice of PROMIS PSCC and reworded items. The reworded items were then tested with the next round of interviewees. This process was meant to enhance the flow, comprehension, and overall clarity of each item in each domain. If necessary, we revised the English version of the item to maintain conceptual equivalence with the Spanish version.

## 4. Results

The participant sociodemographic characteristics for the first round of cognitive interviews are described in Table 1. Table 2 illustrates severity of depression, anxiety, fatigue, and alcohol use among the participants. Although participants understood most of the PROMIS items in Spanish, we did find several items that were difficult to comprehend (Table 4): seven out of 61 anxiety items, 6 out of 48 depression items, 18 out of 42 fatigue items, and 7 out of 44 alcohol use items were found to have poor comprehension.

The anxiety domain (Table 4) had several items that were difficult to comprehend. The words “atemorizado,” “súbitas,” “espasmos,” “indecisos,” and “sobresalté” are not often used in everyday speaking. Double negatives also caused confusion. In the fatigue domain, we had the same problems with difficult words used to explain the concept of fatigue. The population better understood the concept of fatigue by using the word “agotado,” rather than “exhausto.” Also, when making questions to ask “on average” it was best understood when each term was explained in detail rather than using the word “promedio” meaning on average in arithmetic terms. Most participants preferred active voice, detail, and explanation of the concept. The alcohol use subdomain showed the same difficulties with complex words such as “atareado” and “tome,” and no definite translation for the concept of “high.”

The fatigue domain had the most items that were difficult to comprehend. The main problem with comprehension for most items was use of uncommon words, sentence structure, passive voice, and in maintaining consistency of terms. In the depression domain, we found the words “estrecha,” “abrumador,” and “pesimista” to be uncommonly used and unfamiliar to participants. Some participants were confused about the difference between the words “desesperanzado” and “desesperado.” Although similar in sound, they have different meanings: “desesperado,” meaning desperate, is the word most frequently used; “desesperanzado,” meaning hopeless, is a polysyllabic word that confused participants because of its infrequent use. The concept of “emotional exhaustion” was lost when placed in a complex sentence. The use of describing the main concept helped improve comprehension of the item, but it did not help participants to respond to the item.

After we identified these changes, we tested the revised versions once more via cognitive interviewing of five additional participants. These items were refined based on participant feedback; the items were then submitted to the PSCC. After further refinement of items from the PSCC, we tested the items with five additional cognitive interviews. The PSCC translation review team accepted the recommended final changes for difficult to comprehend anxiety, depression, fatigue, and alcohol use items identified in Table 4.

## 5. Discussion

This study explains a process used in conducting cognitive interviews to test and define PROMIS items among Spanish speakers living with HIV. This process allowed for an improvement in comprehension of items and, thus, an improvement in measurement skills. We used an extensive translation process known as the Functional Assessment of Chronic Illness Therapy (FACIT) translation measurement system [5] and afterwards conducted cognitive interviews to identify items that may still cause problems in comprehension. The cognitive interviewing step after the translation of items ensures that items are culturally and linguistically appropriate for the target population. Using this process, we identified items participants had difficulty comprehending (Table 4). Problems with comprehension related mainly to the complexity of the words used. Words containing more than three syllables were difficult to comprehend. Words that sounded like other more commonly used words, such as the case of “desesperado” and “desesperanzado,” were difficult to understand. The term preferred for fatigue is “agotado” rather than “cansado” or “agotado.” Our study population tended to have low levels of formal education and literacy and these factors often impeded comprehension of some of the items; other studies have found that low level of literacy among Spanish speakers is associated with low levels of comprehension in other types of measures [13, 24–26].

Currently, there are resources such as the Lexile Analyzer available in Spanish to determine the reading comprehension level of each item. However, this does not take into account the need to use simple words or ensure that the developer's concept is represented properly. Therefore, it appears that cognitive interviewing may be a necessary step in the translation of items. Although the process is time-intensive, it produces effective results. This study's findings support previous research in which cognitive interviews allowed for the identification of language that was easier to comprehend and identified the need for the inclusion of explanatory phrases to enhance item comprehension [27]. The use of cognitive interviewing in addition to the FACIT translation process provides easier-to-comprehend items and may minimize the language barriers between providers and patients.

Translating health care instruments to other languages is not a simple process. It is often necessary to conduct cognitive interviews with participants from a target community and with a particular condition in order to achieve adequate item comprehension among study participants. Based on the results of cognitive testing, an item may have to be worded differently, use words that are not direct translations, or include phrases that may clarify the content of the item.

Limitations of this study need to be considered. The study is limited by a modest sample size consisting of mostly middle-aged men recruited in two west coast cities: Seattle, Washington, and San Diego, California. Future studies should seek to (1) have more gender diversity (include more women), (2) have age diversity, and (3) include participants from a wider array of literacy levels (include more native Spanish speakers with a higher literacy level). Due to the location of this study, most of the Spanish-speaking participants were of Mexican descent; future studies would benefit from including participants from other Latin American countries.

This study used cognitive interviews to evaluate PROMIS item comprehension for depression, anxiety, fatigue, and alcohol use among Spanish speakers living with HIV. Using this process allowed for improvements in comprehension of items for these domains. For the next step of this research, a need exists to use quantitative methods to ensure measurement equivalence between the Spanish and English versions of the PROMIS item banks, as done in previous studies [28, 29]. Such research will require the collection of a large number of completed PROMIS items in Spanish and English.

## Figures and Tables

**Table 1:** Characteristics of the participant population for the first round of cognitive interviews.

Characteristics	Depression domain (*n* = 13)	Anxiety domain (*n* = 9)	Fatigue domain (*n* = 15)	Alcohol Use domain (*n* = 19)	Total (*n* = 56)
Gender					
Male	10 (77%)	6 (67%)	11 (73%)	14 (74%)	41 (73%)
Female	3 (23%)	3 (33%)	4 (27%)	5 (26%)	15 (27%)
Place of birth					
US	1 (8%)	0 (0%)	0 (0%)	2 (11%)	3 (5%)
Mexico	9 (69%)	7 (78%)	0 (0%)	17 (89%)	33 (59%)
Other	3 (23%)	2 (22%)	0 (0%)	0 (0%)	5 (9%)
Unknown	0 (0%)	0 (0%)	15 (100%)	0 (0%)	15 (27%)
Age (yrs)					
18–29	0 (0%)	1 (11%)	1 (7%)	5 (26%)	7 (13%)
30–39	1 (8%)	2 (22%)	1 (7%)	4 (21%)	8 (14%)
40–49	7 (54%)	3 (33%)	8 (53%)	10 (53%)	28 (50%)
50–59	3 (23%)	3 (33%)	2 (13%)	0 (0%)	8 (14%)
≥60	2 (15%)	0 (0%)	3 (20%)	0 (0%)	5 (9%)
Most recent CD-4 Cell count (mm^3^)					
0–199	4 (31%)	2 (22%)	0 (0%)	1 (5%)	7 (13%)
200–349	5 (38%)	2 (22%)	0 (0%)	1 (5%)	8 (14%)
≥350	4 (31%)	5 (56%)	15 (100%)	14 (74%)	38 (68%)
Unknown	0 (0%)	0 (0%)	0 (0%)	3 (16%)	3 (5%)
Time since HIV Diagnosis (yrs)					
0–9	4 (31%)	2 (22%)	3 (20%)	11 (58%)	20 (36%)
10–19	7 (54%)	5 (56%)	10 (67%)	6 (32%)	28 (50%)
20–30	2 (15%)	1 (11%)	2 (13%)	1 (5%)	6 (11%)
Unknown	0 (0%)	1 (11%)	0 (0%)	1 (5%)	2 (4%)
HIV transmission risk factor					
MSM^1^	7 (54%)	5 (56%)	10 (67%)	8 (42%)	30 (54%)
IVDU^2^	0 (0%)	1 (11%)	2 (13%)	0 (0%)	3 (5%)
Heterosexual	4 (31%)	3 (33%)	3 (20%)	2 (11%)	12 (21%)
Other	2 (16%)	0 (0%)	0 (0%)	0 (0%)	2 (4%)
Unknown	0 (0%)	0 (0%)	0 (0%)	9 (47%)	9 (16%)

^1^MSM: defined as men who have sex with men.

^2^IVDU: defined as intravenous drug user.

**Table 2:** Severity of disease found among participants in the first round of cognitive interviews.

	*n*	%
Depression domain *n* = 13 (PHQ-9)		
Mild	6	46
Moderate	5	38
Severe	2	15
Anxiety domain *n* = 9 (PHQ-5)		
Anxiety	5	56
Anxiety with panic	4	44
Fatigue domain *n* = 15 (HIV symptom scale)		
Mild	5	33%
Moderate/severe	10	67%
Alcohol use domain *n* = 19 (AUDIT-C)		
Mild/moderate (4–7)	5	26
Severe (8–13)	14	74

**Table 3:** The cognitive interview guide for participants.

English guide	
(1) Can you tell me in your own words what the question/statement means to you? (2) What were you thinking of when you answered the question/statement? (3) Were there any words in the question/statement that were not clear? Could it be reworded? (4) How did you go about deciding on which answer to pick? (5) Was the question/statement easy/hard/OK to answer for the past 7 days/past 30 days/past 4 weeks (corresponds to time frame specified in domain)? (6) How did you choose between some of the answer choices, for example “Rarely” and “Sometimes” or “Often” and “Always”?	

Spanish guide	
(1) Podría decirme en sus propias palabras, qué significa para Ud. la siguiente pregunta o afirmación? (2) En qué estaba pensando, cuando contestó esta pregunta o afirmación? (3) Existe(n) alguna(s) palabra(s) en la pregunta o afirmación qué no estaba(n) claras? Podría escribirse de otra manera? (4) Cómo decidió qué respuesta escoger? (5) Fue la pregunta o afirmación fácil/difícil/o sin problema para contestar sobre los últimos 7 días/30 días/4 semanas? (6) Cómo escogió Ud. entre algunas de las opciones para contestar, por ejemplo entre: “Rara vez”, “A veces”, “A menudo,” y “Siempre”?	

**Table 4:** PROMIS items with their corresponding Spanish counterparts, revised items in italics.

Item number	Item in English	Item in Spanish (preapproved)	Reason for problem with comprehension	Item in Spanish (approved)
Depression domain items				
1	In the last 7 days, I felt helpless	En los últimos 7 días, me sentí indefenso/a	Participants assumed that “indefenso/a” was related to the ability to speak English	*En los últimos 7 días, me sentí indefenso/a (que no podía hacer nada para ayudarme).*
2	In the last 7 days, I had trouble feeling close to people	En los últimos 7 días, me costó trabajo sentir una relación *estrecha* con la gente	Participants did not know the meaning of the word “estrecha”	*En los últimos 7 días, me costó trabajo sentir una relación estrecha (cercana) con la gente*
3	In the last 7 days, I found that things in my life were overwhelming	En los últimos 7 días, descubrí que había cosas en mi vida que eran *abrumadoras*	Participants did not know the meaning of the word “abrumadoras”	*En los últimos 7 días, descubrí que había cosas en mi vida que eran demasiado para mí*
4	In the last 7 days, I felt hopeless	En los últimos 7 días, me sentí *desesperanzado/a*	Participants confused the word “desesperanzado/a” with “desesperado/a”	*En los últimos 7 días, sentí que no tenía esperanza*
5	In the last 7 days, I felt pessimistic	En los últimos 7 días, me sentí *pesimista*	Participants did not know the meaning of the word “pesimista”	*En los últimos 7 días, me sentí pesimista (que veía las cosas de modo negativo)*
6	In the last 7 days, I felt emotionally exhausted	En los últimos 7 días, me sentí *exhausto/a emocionalmente*	Participants thought that “exhuasto/a emocionalmente” referred to a physical act and did not understand the relationship to an emotion	*En los últimos 7 días, me sentí completamente agotado emocionalmente*

Anxiety domain items				
1	In the last 7 days, I felt frightened	En los últimos 7 días, me sentí *atemorizado/a*	Participants did not know the meaning of the word “atemorizado/a”	*En los últimos 7 días, sentí mucho temor*
2	In the last 7 days, I had sudden feelings of panic	En los últimos 7 días, tuve sensaciones *súbitas* de pánico	Participants did not know the meaning of the word “súbita”	*En los últimos 7 días, tuve sensaciones de pánico repentinas*
3	In the last 7 days, I was easily startled	En los últimos 7 días, me *sobresalté* fácilmente	Participants were not comfortable using the word “sobresalté” in regards to a person; they thought it would be used with an inanimate object	*En los últimos 7 días, me sobresalté (asusté) fácilmente*
4	In the last 7 days, I found it hard to focus on anything other than my anxiety	En los últimos 7 días, tuve dificultad para concentrarme *en nada que no fuera* mi ansiedad	Participants did not comprehend the sentence because of the use of the double negative “nada” and “que no fuera.”	*En los últimos 7 días, tuve dificultad para concentrarme en otra cosa que no fuera mi ansiedad*
5	In the last 7 days, my worries overwhelmed me	En los últimos 7 días, sentí que mis inquietudes me *abrumarón*	Participants did not understand the meaning of the word “abrumaron”	*En los últimos 7 días, mis inquietudes fueron demasiado para mí*
6	In the last 7 days, I had twitching or trembling muscles	En los últimos 7 días, tuve *espasmos* o temblores musculares	Participants did not know the meaning of the word “espasmos”	*En los últimos 7 días, mis músculos temblaron o se movieron solos*
7	In the last 7 days, I felt indecisive	En los últimos 7 días, me sentí *indeciso/a*	Participants did not know the meaning of the word “indeciso/a”	*En los últimos 7 días, me sentí indeciso/a (que tenía dificultad para tomar decisiones)*

Fatigue domain items				
1	In the past 7 days, how often were you physically drained?	En los últimos 7 días, con qué frecuencia se sintió consumido/a físicamente?	Participants did not understand the meaning of the word “consumido/a.”	* En los últimos 7 días, con qué frecuencia se sintió consumido/a (desgastado/a) físicamente?*
2	In the past 7 days, how often did your fatigue make you slowed down your thinking?	En los últimos 7 días, con qué frecuencia el agotamiento le hizo *sentir que pensaba* con más lentitud?	Participants did not understand the concept of the word; the sentence structure needed to be changed for the concept to be understood.	*En los últimos 7 días, con qué frecuencia el agotamiento le hizo pensar con más lentitud?*
3	In the past 7 days, how often did you have to push yourself to get things done because of your fatigue?	En los últimos 7 días, con qué frecuencia se tuvo que forzar para hacer sus actividades debido al agotamiento?	Participants did not understand the concept of the word; the sentence structure needed to be changed for the concept to be understood.	*En los últimos 7 días, con qué frecuencia se tuvo que forzar para completar sus actividades debido al agotamiento?*
4	In the past 7 days, how often did you have to limit your social activities because of your fatigue?	En los últimos 7 días, con qué frecuencia el agotamiento interfirió en su capacidad de participar en *actividades recreativas*?	Participants did not understand the concept and more adjectives needed to be added to be more specific and for the concept to be understood.	*En los últimos 7 días, con qué frecuencia el agotamiento interfirió en su capacidad de participar en actividades físicas recreativas?*
5	In the past 7 days, how much were you bothered by your fatigue on average?	En los últimos 7 días, qué tan rendido/a se sintió *en promedio*?	Participants did not understand the concept of “on average” as a direct translation to “promedio.” The sentence structure needed to be changed for the concept to be understood.	*En los últimos 7 días, qué tan frecuente se sintió rendido?*
6	In the past 7 days, how run-down did you feel on average?	En los últimos 7 días, qué tan agotado/a estuvo *en promedio*?	Participants did not understand the concept of “on average” as a direct translation to “promedio.” The sentence structure needed to be changed for the concept to be understood.	*En los últimos 7 días, qué tan frecuente se sintió agotado/a?*
7	In the past 7 days, how often did you experience extreme exhaustion?	En los últimos 7 días, con qué frecuencia sintió *extenuación extrema*?	Participants did not understand the meaning of the word “extenuación.”	*En los últimos 7 días, con qué frecuencia se sintió exhausto/a en un grado extremo?*
8	In the past 7 days, how often were you too exhausted to chew and swallow food?	En los últimos 7 días, con qué frecuencia se ha sentido *demasiado cansado* para masticar y tragar la comida?	Participants did not understand the reasoning for the question, if it referred to feeling, but still doing the action or just having the feeling of exhaustion which prevented them from eating.	*En los últimos 7 días, con qué frecuencia estuvo tan agotado/a que no pudo masticar y tragar la comida?*
9	In the past 7 days, how often did you wake up feeling exhausted?	En los últimos 7 días, con qué frecuencia se *ha despertado* sintiéndose exhausto?	Needed to use active voice for the concept to be understood.	*En los últimos 7 días, con qué frecuencia se despertó sintiéndose exhausto/a?*
10	In the past 7 days, how much often did you feel so exhausted that you stayed in bed all day?	En los últimos 7 días, con qué frecuencia se ha sentido tan exhausto que se quedó en la cama todo el día?	Needed to use active voice for the concept to be understood.	*En los últimos 7 días, con qué frecuencia estuvo tan exhausto/a que se quedó todo el día en cama? *
11	In the past 7 days, how often were you too exhausted to take your medication?	En los últimos 7 días, con qué frecuencia *se ha sentido demasiado cansado* como para tomar la medicina?	Needed to use active voice for the concept to be understood and also to be consistent with the term “exhausto” rather than “cansado.”	* En los últimos 7 días, con qué frecuencia estuvo tan agotado/a que no pudo tomar su medicina?*
12	In the past 7 days, how often were you too exhausted to carry out your daily responsibilities?	En los últimos 7 días, con qué frecuencia *se ha sentido demasiado cansado *como para cumplir con sus responsabilidades diarias?	Needed to use active voice for the concept to be understood and also to be consistent with the term “exhausto” rather than “cansado.”	*En los últimos 7 días, con qué frecuencia estuvo tan agotado/a que no pudo cumplir con sus responsabilidades diarias? *
13	In the past 7 days, how often did your body feel exhausted?	En los últimos 7 días, con qué frecuencia se ha sentido que su cuerpo estaba exhausto?	Needed to use active voice for the concept to be understood.	*En los últimos 7 días, con qué frecuencia sintió que su cuerpo estaba exhausto?*
14	In the past 7 days, how often were you so tired that you made more mistakes than usual?	En los últimos 7 días, con qué frecuencia *ha cometido más errores de lo habitual debido a su cansancio*?	Needed to use active voice for the concept to be understood.	*En los últimos 7 días, con qué frecuencia estuvo tan cansado/a que cometió más errores de lo habitual? *
15	In the past 7 days, how often were you so exhausted that you missed appointments?	En los últimos 7 días, *con qué frecuencia ha perdido las citas porque se sintió cansado*?	Needed to use active voice for the concept to be understood.	*En los últimos 7 días, con qué frecuencia estuvo tan exhausto/a que no pudo acudir a sus citas?*
16	In the past 7 days, how often were you so exhausted that everything took more effort than usual?	En los últimos 7 días, con qué frecuencia ha estado tan cansado *que cualquier tarea le llevo más esfuerzo que lo habitual*?	Changed the sentence structure and kept the same terminology and more specific wording.	*En los últimos 7 días, con qué frecuencia estuvo tan exhausto/a que todo requirió más esfuerzo de lo habitual?*
17	In the past 7 days, how often were you too exhausted to spend time with other people?	En los últimos 7 días, con qué frecuencia *se ha sentido tan cansado que evitó *la compañía de otras personas?	Changed the wording in the sentence. Most participants did not find using avoidance (evitó) as something they would do if they felt exhausted and preferred using, not able to be around other people.	*En los últimos 7 días, con qué frecuencia estuvo tan agotado/a que no pudo estar en compañía de otras personas?*
18	In the past 7 days, how often were you too exhausted to concentrate?	En los últimos 7 días, *con qué frecuencia se ha sentido demasiado cansado* como para concentrarse?	Participants preferred the use of the same terminology.	*En los últimos 7 días, con qué frecuencia estuvo tan agotado/a que no pudo concentrarse? *

Alcohol use subdomain				
1	In the past 30 days, I finished several drinks fast to get a quick effect.	En los últimos 30 días, terminé varias *bebidas con prisa* para sentir un efecto más rápido.	Participants did not understand the concept of finished drinking more than one drink at a point in time, unless the word “tomé” was used.	*En los últimos 30 días, tomé varias bebidas muy deprisa para sentir un efecto más rápido.*
2	In the past 30 days, I drank heavily at a single sitting.	En los últimos 30 días, *tomé bastante* en una sola sesión OR En los últimos 30 días, *tome bastante* en una sola sentada	Participants had a hard time understanding the concept; the developing team also had a difficult time at coming to a decision in regard to drinking heavily at one point in time.	*En los últimos 30 días, tomé mucho en una sola sesión. *
3	In the past 30 days, I used alcohol and other drugs together, to get high	En los últimos 30 días, *use drogas y alcohol juntos para intoxicarme*.	Participants agreed that “intoxicarme” implies suffering from intoxication or drug overdose, not to get a feeling of being high.	*En los últimos 30 días, tomaba alcohol y drogas juntos para sentir más el efecto (“high”).*
4	In the past 30 days, I drank because I was irritable.	En los últimos 30 días, tomé porque estaba *irritable* (me enfade fácilmente).	Participants felt “enfade” was a word only used in certain parts of Mexico and was not understood by all Spanish-speaking participants.	*En los últimos 30 días, tomé porque me sentía irritado/a*
5	In the past 30 days, I drank at the end of a busy day.	En los últimos 30 días, tomé al final de un día *atareado*.	Atareado was a more complex word than ocupado; simpler terms helped participants understand the concept clearly.	*En los últimos 30 días, tomé al final de un día en que estuve muy ocupado/a*
6	In the past 30 days, I drank to avoid my problems.	En los últimos 30 días, tome para *evitar mis problemas*.	Participants did not feel the word “evitar” could be applied to a concept but rather preferred it would be applied to concrete items.	*En los últimos 30 días, tomé para huir de mis problemas.*
7	In the past 30 days, I was worried about how drinking was affecting my health.	En los últimos 30 días, me preocupé sobre como la bebida *afectaba* mi salud.	Participants preferred the item to be worded in past perfect tense.	*En los últimos 30 días, estaba preocupado/a por cómo la bebida estaba afectando mi salud. *
